# Navigating structural demands and relational care: a qualitative study of public health nurses’ experiences and handling of online screening tools

**DOI:** 10.1186/s12912-026-04727-4

**Published:** 2026-05-07

**Authors:** Trine Holm, Elin Thygesen, Geir Inge Hausvik, Thomas Westergren

**Affiliations:** 1 Department of Health and Nursing Science, University of Agder, Jon Lillistons vei 9, Grimstad, 4879 Norway; 2 Department of Information Systems, University of Agder, Kristiansand, Norway; 3 Department of Public Health at the University of Stavanger, University of Stavanger, Stavanger, Norway; 4 Department of Health and Nursing Science and academic leader of the Center for e-Health at the University of Agder, Kristiansand, Norway

**Keywords:** Online screening tools, Primary health care, Public health nursing, Well child care

## Abstract

**Background:**

Online screening tools are recommended in well-child visits to support early detection and standardized assessment, while also facilitating meaningful dialogue and collaboration with parents. However, little is known about how public health nurses experience and handle these tools in practice. The aim of this study was to explore public health nurses’ experiences with, and handling of, online screening tools in their work practices in well-child clinics and school health services.

**Method:**

We used observations and semi-structured interviews to capture public health nurses’ experiences and practices after integration of online screening tools. Public health nurses were observed during well-child visits, and when possible, also before and after visits to provide a broader understanding of their practice. In total, eight public health nurses were observed in connection to well-child visits with families of children aged 0–7 years. Interviews were conducted over an extended time-period, with a total of 13 public health nurses participating individually or in groups. Data was analyzed using reflexive thematic analysis of verbatim transcripts and field notes.

**Findings:**

The findings from this study illustrate how public health nurses experienced tension between structural requirements and relational values when online screening tools were implemented into their practice. The relationship with families was considered the cornerstone of their work, shaping trust, continuity, and holistic understanding. While the tools offered opportunities for structured assessment and dialogue, they also introduced uncertainty about interpretation, follow-up, and professional responsibility, often diverting time and attention away from nurturing relationships. The experienced tension between standardized procedures and relational care reflected a deeper struggle to balance professional obligations with the relational connection that public health nurses regarded as foundational to their role. These dynamics are explored through three themes: When the Structures Comes Between Us, When We Get Trapped Within the Structures, and When the Structures Supports Our Practice.

**Conclusion:**

To realize the potential benefits of online screening tools, integration must safeguard both structural requirements and relational needs, ensuring that structures do not come at the expense of trust, relationship, and presence.

**Trial registration:**

Not applicable.

**Supplementary Information:**

The online version contains supplementary material available at 10.1186/s12912-026-04727-4.

## Background

Primary health care personnel play a key role in safeguarding children’s health and development, through supervision of parents, examination and observation of child’s health and development, and by coordinating care. Structured screening tools are widely recognized as promising instruments for strengthening this work because they enable systematic assessment and early identification of developmental and psychological concerns [1, 2]. Use of such tools may, however, challenge competence and imply practice change which are sparsely understood in this clinical field [3]. Beyond training, new routines, and implementation; moving between more experience-based dialogue and assessment in health promotive work ─ as within the tradition of Scandinavian primary child and school health care [1] ─ and utilizing and interpreting structured client-reported information requires comprehensive understanding of benefits and pitfalls between those modes [3].

Evidence suggests that such tools can improve identification rates compared to clinical judgment alone [4], support health-promoting dialogues, and enhance collaboration with parents and strengthen user involvement [5, 6]. These opportunities are particularly important given that early childhood is a sensitive period in neurodevelopment, where timely intervention can have long-term benefits for children, families, and society [7–9].

Despite these potential benefits, the routine use of structured screening tools in primary health care is not consistently implemented across countries. In many contexts, assessments still rely primarily on clinical observation and parental concerns rather than standardized procedures [1, 10]. This lack of standardization may lead to inconsistent documentation and missed opportunities for early intervention. While international guidelines recommend routine screening, implementation studies highlight significant barriers, including time constraints, insufficient training, and unclear follow-up procedures [6]. In addition, studies indicate that health care personnel often report limited competence in addressing psychosocial and mental health concerns, expressing uncertainty about how to interpret screening results and follow-up with families [3]. A central part of addressing these challenges is ensuring sufficient training and establishing clear plans for follow-up [11]. However, these challenges raise questions about how screening tools can be integrated into everyday practice without compromising relational work, which is central to health care personnels’ role [12].

Digitalization of screening tools is often presented as a solution to technical and organizational barriers, offering improved usability, cost-effectiveness, and reduced interpretation errors [13, 14]. However, online tools introduce new complexities, such as varying digital competencies, cultural adaptation issues, and persistent uncertainty about interpreting and communicating results [6, 11]. As a result, health care personnel must navigate these digital demands while sustaining trust-based relationships with families. Simultaneously, research shows that parents value open dialogue about screening outcomes and prefer discussions that emphasize their child’s strengths and areas of concern rather than simply receiving a score or result [15–17]. These expectations highlight the relational dimension of screening and raise questions about how health care personnel balance structured assessments with individualized, trust-based communication.

Although various studies have explored the integration of standardized screening within pediatric primary health care, the understanding of how these innovations specifically influence health care personnel’ practices and their ability to engage relationally remains limited [11]. Much of the existing research has focused on implementation barriers and facilitators, addressing the process and how to structurally integrate the science into practice [18]. This is leaving an insufficient exploration post implementation, into how health care personnel experience and navigate the tension between structural demands and the necessity for building relationships with children and families. This shows the need for a deeper understanding of how health care personnel navigate the structural and relational demands involved in the use of online screening tools in primary health care. Exploring how they interpret and integrate these tools into practice can reveal their impact on routines, decision-making, collaboration, and family interactions. Therefore, we aim to explore this matter among public health nurses (PHNs) working with children aged 0–7 years in well-child clinics and school health services through the following research question:

How do PHNs experience and manage the use of online screening tools in their work practices?

## Method

### Context

In Norway, well-child clinics and school health services are key components of children’s preventive and health-promoting care, run by municipality primary health care services. These services provide free, universally accessible follow-up from birth to age 16, including 14 scheduled well-child visits for children before the age of six. The services are led by public health nurses (PHNs) who are mandated to monitor children’s physical, psychological, and social development, identify concerns at an early stage, offer guidance and support to families, and collaborate with other services when needed [19].

Although routine use of screening tools during well-child visits is recommended in several countries, these recommendations is not embedded in Norway’s national guidelines [19]. In Norwegian child health services, validated screening tools are largely confined to research contexts, and assessments typically rely on clinical observation and parental concerns [1]. To address this gap and explore how systematic screening could be integrated into everyday practice, *Starting Right* implemented an online screening platform in well-child clinics and school health services. The aim was to support early identification and health-promoting dialogue between PHNs and families, and to streamline service processes in child health clinics and school health services [20].

The platform included validated questionnaires, referred to in this article as online screening tools, covering various dimensions of child health and development. License for all questionnaires in clinical work was handled by CheckWare Ltd. and this qualitative study did not require any further permission from license owners, as we did not analyze or use questionnaire data.

**Fig. 1:**
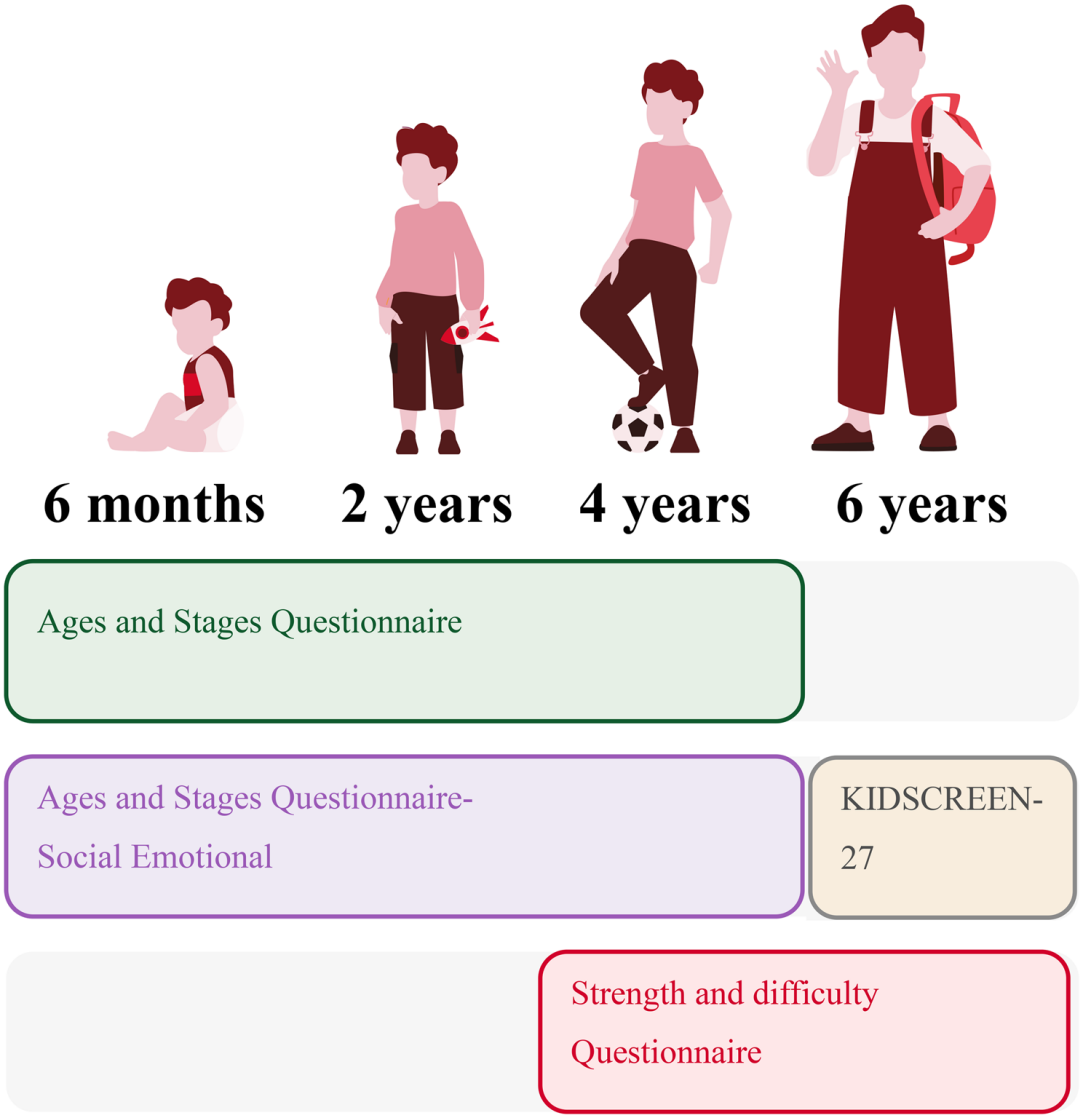
Overview of Screening Tools Across Age Groups. The instruments include measures of general development (Ages and Stages Questionnaire), social–emotional development (Ages and Stages Questionnaire–Social Emotional), general mental health (Strengths and Difficulties Questionnaire), and health-related quality of life (KIDSCREEN-27)

Eight municipalities in southern Norway participated at the time of the study, and each retained the flexibility to decide which tools to use and for which age groups. Figure 1 shows the questionnaires available for each age group, and which were made available based on stakeholders’ advice in accordance with national guidelines and license agreements by CheckWare Ltd.

Prior to implementation, PHNs received training in both the questionnaires (5 h) and the digital platform (3 h) [20]. During the pilot phase, a medical secretary supported the distribution of questionnaires to parents ahead of scheduled well-child visits. This facilitation contributed to increased adoption among PHNs and was perceived as satisfactory by PHNs [20]. In addition, annual staff training days were held for all staff using the online screening tools. PHNs also participated in regular meetings with the project group that included case discussions, and PHNs had access to daily technical support.

**Fig. 2:**
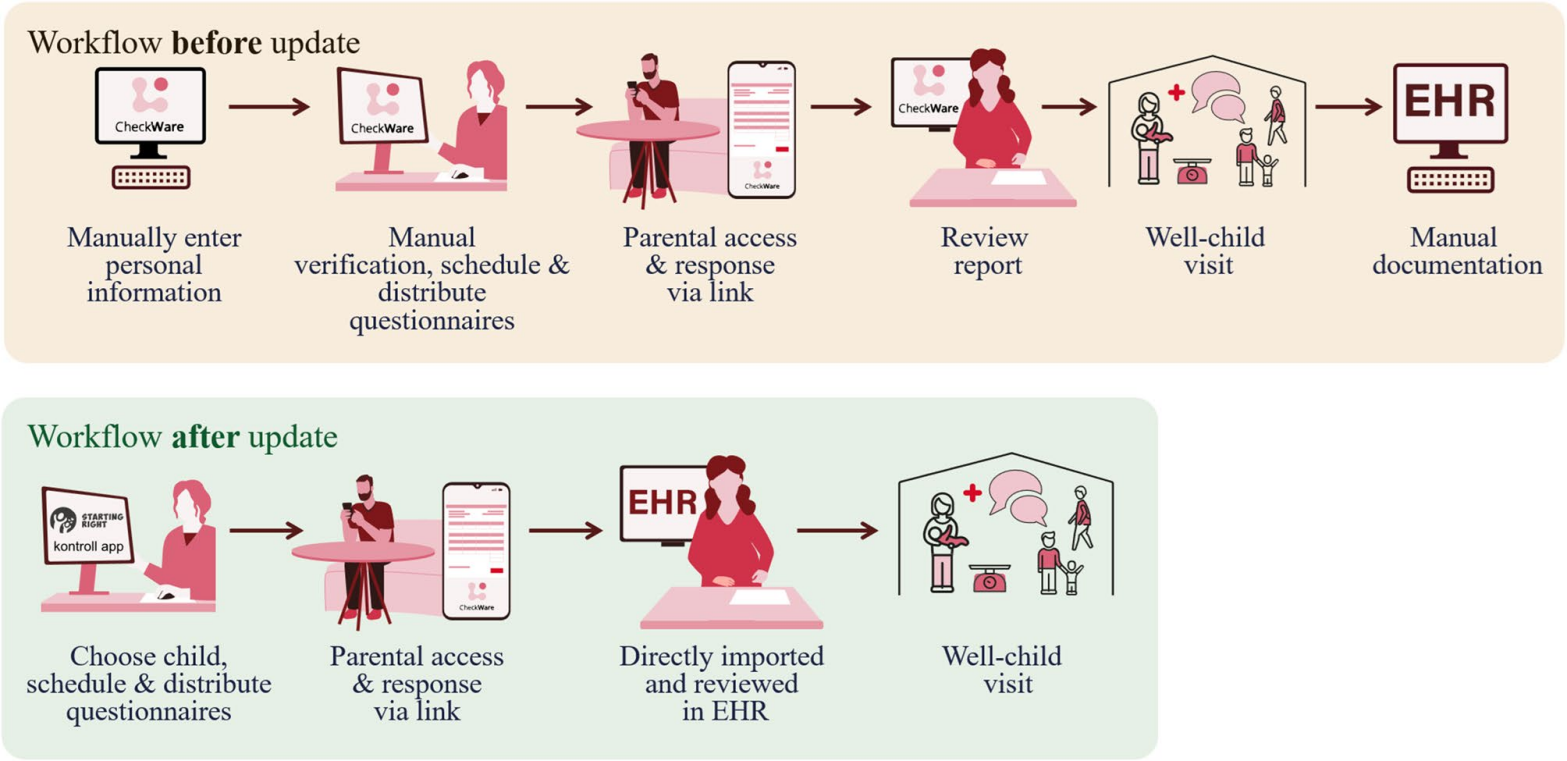
Illustrates the workflow before and after platform update. Workflow changed from manual handling in CheckWare Ltd. (Trondheim, Norway) to an integrated Starting Right control app (developed by Egde AS, Kristiansand, Norway) application with automatic retrieval of parent information and direct electronic health record import

During the study period, the digital platform used for distributing and collecting questionnaire reports underwent a structural change designed to streamline the process for PHNs. The modification made the workflow simpler and more efficient (See Fig. 2).

### Study design

This study, consisting of observations and interviews, is rooted in a qualitative design informed by phenomenological, hermeneutic, and existential perspectives, drawing particularly on the philosophical framework outlined by Dahlberg and Dahlberg [21]. Central to this approach is the understanding that human experience is fundamentally intertwined with the world in which it occurs. Knowledge is therefore seen as emerging through close engagement with the field, and the research process is both descriptive and interpretive in nature. Reflexivity was employed throughout the study to ensure credibility and transparency.

Rather than treating subjectivity and objectivity as mutually exclusive concepts, the study acknowledges their dynamic interplay [21]. The researchers’ interpretations were shaped by both the empirical material and their own theoretical and contextual positioning. The research team brought together interdisciplinary expertise from nursing and information systems. Two of the authors (ET, GIH) were affiliated with a center for e-health research, while TW had previously led the Starting Right project. Data collection was carried out by TH who were involved in project meetings related to *Starting Right*. None of the researchers had experience with using online screening tools in well-child clinics either as clinicians or parents. Methodological coherence was maintained by ensuring alignment between the study’s theoretical underpinnings, research questions, and design. The reporting adheres to the Standards for Reporting Qualitative Research (SRQR) as proposed by O’Brienet al. [22].

### Participants and recruitment

A total of 13 PHNs from three municipalities, representing five well-child clinics and two school health services, agreed to participate in the study. PHNs were recruited through leaders of the well-child clinic and school health service involved in the *Starting Right* initiative. These leaders shared study information with PHNs, who then contacted the first author to express interest and arrange data collection.

We collected data from 12 interviews and from observations during 20 well-child visits. Prior to each visit, parents were informed about the study both verbally and in writing, and 23 parents provided written consent to participate alongside their children. While this article focuses on PHNs’ practices during well-child visits, parents’ experiences and perspectives are reported in another article [17]. Participants’ involvement in data collections activities is presented in Table 1, and characteristics are presented in Table 2.

**Table 1:** Participants involved in data collection

Participant	Interviewed May- December 2022	Interviewed August- December 2024	Observed
Participant 001	X		X
Participant 002	X		X
Participant 003	X	X	X
Participant 004	X		X
Participant 005	X		X
Participant 006	X		
Participant 007	X		X
Participant 008	X	X	X
Participant 009	X	X	
Participant 010	X		X
Participant 011		X	
Participant 012		X	
Participant 013		X	

**Table 2:** Participants characteristics

Characteristics	PHNs interviewed (*n* = 13)	PHNs observed (*n* = 8)
**Age**,** years (min**,** max)**	46 (27, 62)	50 (27, 62)
**Years of experience as a PHN (min**,** max)**	10 (2, 26)	10,5 (2, 26)
**Experience with questionnaire**		
SDQ, n	5	3
KIDSCREEN, n	4	3
ASQ-SE, n	8	5
ASQ, n	8	5

Abbreviations: PHN: Public health nurse, ASQ: Ages & Stages Questionnaire, ASQ-SE: Ages & Stages Questionnaire, Social–Emotional, SDQ: Strength & Difficulties Questionnaire

### Data collection

We selected qualitative methods, including field observations and interviews, to explore PHNs´ experiences and handling of online screening tools in their work practice. Data collections were conducted at two different time periods; observations and the first round of interviews were conducted between May and December 2022 and follow up interviews took place between August and December 2024.

### Observations

Observations were employed to gain contextual understanding of clinical practice and to capture both the actions and reflections of PHNs. The first author followed eight PHNs throughout various stages of a well-child visit, before, during, and after, to document their practices and interactions. An observation guide ensured systematic and consistent data collection, providing a structured framework for observing workflow, communication, and handling and assessment of screening information (see supplementary file 1). The observer adopted an observer-as-participant stance [23], maintaining a primarily observational role during the well-child visits and speaking only when it occurred naturally or when addressed by the PHN, parents, or child, to minimize influence on the well-child visits. This approach balanced limited engagement with a deliberate background position to minimize influence on the encounters. In addition, the observation guide was complemented by a think-aloud technique in the preparatory and follow-up phases when feasible, enabling PHNs to articulate their reflections and decision-making processes. If pauses occurred, the observer used brief think-aloud prompts to encourage the participant to continue verbalizing their thoughts. This technique was valuable for accessing cognitive processes that might otherwise remain implicit [24]. These sessions were audio-recorded and transcribed.

To avoid disrupting the clinical setting and ensure a natural and safe interaction, the think-aloud technique was not used during the well-child visits. Instead, brief field notes were taken and later expanded into detailed observational notes categorized according to Hammersley and Atkinson [23] framework, distinguishing between methodological notes, descriptive data, and reflective commentary. This approach was applied both to the three PHNs observed throughout the entire process and to additional five PHNs who were only observed during the well-child visit because of practical constraints of PHN’s daily work routines.

### Interviews

Interviews were chosen to allow for in-depth exploration of PHNs’ experiences, reflections, and contextual nuances that would not be fully captured through observation alone. The interviews included individual interviews (*n* = 10), one paired interview, and a group interview with four PHNs. Ten PHNs were interviewed before the implementation of an updated version of the tool. Three of them were interviewed twice, before and after the implementation, and an additional three were interviewed only after the implementation, to capture changes over time. All interviews were conducted in the PHNs’ offices, lasting between 30 and 58 min and followed a semi-structured interview guide to ensure that key topics were covered while allowing participants to elaborate on their experiences. The interview guide focused on PHNs’ experiences with the screening tools, including changes in their work process, the relevance of the content, the assessment process, and impact on the well-child visit (see supplementary file 1). Follow-up questions were asked to obtain supplementary reflections from the PHNs.

### Analysis

Transcripts of interviews and observations were analyzed in conjunction with observation notes inspired by reflexive thematic analysis, following the six-phase approach outlined by Braun and Clarke [25]. This approach promotes a dynamic and interactive approach between the phases. The first author led the analytical process, enabling a reflective approach to the data. The co-authors contributed valuable insights and reflections by reviewing the coding process, the thematic structure, and drafts of the findings. Together, they assessed the analytical results by posing critical questions to ensure robust and thorough analysis.

Familiarization with the data began during data collection, as the first author wrote reflective notes after each interview and observation. This continued throughout the transcription process and through repeated readings of the material prior to systematic coding. During this phase, the first author also noted emerging patterns and potential preliminary themes. All interview and observational data were organized and coded in a shared codebook using NVivo11 [26]. Initially, coding was conducted using language directly derived from the participants to maintain closeness to the original data. Subsequently, codes were reviewed, refined, and merged where appropriate. The themes were constructed through a latent-level analysis, focusing on underlying meanings and assumptions reflected in participant’s expressed experiences and perspectives. These themes were iteratively reviewed to ensure internal coherence and consistency across the dataset. Once finalized, the first author revisited the entire dataset to confirm that the themes accurately reflected the data as a whole. Finally, each theme was clearly defined and named to encapsulate its central meaning, culminating in the writing up of the analytical findings and the overall report [25]. Quotations from the participants have been translated from Norwegian to English.

### Findings

PHNs in this study emphasized the importance of trust as the foundation for families to share experiences and challenges, and for PHNs to provide relevant guidance. Preserving an open and trusting relationship formed the foundation for how they experienced online screening tools. Simultaneously, their experiences were understood as reflecting a service increasingly shaped by demands for efficiency, standardization, and external definitions of good practice.

Although our initial aim was to explore experiences with online screening tools, our analysis highlighted that tensions related to structural and controlling demands were already embedded in the service, and that the introduction of online tools was experienced to increase these existing dynamics. PHNs described an ambivalent relationship with the tools: they were seen as something that could take focus away from the relational meeting with families, as participant 011 emphasized: “*I worry that if we rely too much on forms*,* people just stop thinking for themselves. Then you lose some of that connection*,* and it all just becomes paperwork. We need to see the person behind it”.* On the other hand, the screening tools were appreciated for their ability to structure assessments, systematize observations, and make children’s needs more visible in dialogue with parents, as illustrated by participant 008: *“There are a few more concrete questions that the parents have answered themselves*,* which we can then explore further. (…) It made her (the mother) reflect on her answers*,* which is an important goal in the guidance process.”*

This tension, between structure and relationship, runs through the material, revealing an ongoing struggle to reconcile structural obligations with the relational foundation of practice while navigating competing demands in daily work. It is explored in two themes highlighting experiences of unresolved tensions “*When the Structure Comes Between Us”* and *“When We Get Trapped Within the Structures”.* The third theme covers experienced opportunities for resolving tensions: “*When the Structure Supports Our Practice”.*

### When the structures come between us

Through interviews and observations, it emerged that PHNs experienced the screening tools coming between them and the families they worked with. They talked about two main concerns; first, that time spent on structural and administrative tasks reduced the time available for relational work, and second, that the screening itself might undermine trust and connection. This theme draws attention to the ongoing negotiation between system-imposed structures and relational care, and how professional judgment and care practices were shaped within institutional boundaries.

In the early phase of the project, PHNs experienced the online screening tools as cumbersome, time-consuming, and poorly integrated into their daily workflow. One key issue was that the tools required access to a separate system, which led to double documentation and lacked notification features. During the project period, the system was updated to include alerts when parents completed, and responses were automatically imported into the patient’s record. PHNs described this as a major improvement. As participant 011 explained: *“Now it goes straight into the child’s record when parents complete the Ages and Stages Questionnaire. We don’t have to log in separately to pull it up anymore*,* so that saves us some time.”*

Despite technical improvements, the screening was not always completed by parents, and the information provided was often incomplete. PHNs expressed that the screening did not cover the totality of elements of the national guidelines, which forced them to prioritize what to emphasize during well-child visits. Observations reflected different strategies: some reviewed the screening together with the family, while others supplemented with additional questions to meet the guideline requirements. In interviews, PHNs wished the screening to cover all guideline topics, assuming the guidelines to be more holistic than questionnaires, so that well-child visits could focus on parents’ concerns and relationship-building rather than rigid checklists. They expected the screening to function as a checklist, reducing pressure from the guideline’s “must do”. PHNs expressed an underlying tension between standardization and individualization: PHNs wanted online tools that freed time for dialogue and professional judgment, not tools that restricted the conversation to fixed control points. As participant 006 put it:

It would have been helpful if they had filled in things like sleep, diet, or other challenges beforehand. Then we could plan the conversation to focus on those challenges, because then we can see them and we wouldn’t have to ask about everything else.

In addition to the extra time required to administer and process information from the screening, PHNs were concerned that the screening itself might create unnecessary strain for some families and, in turn, damage the relationship. Participant 011 explained:

Those we know are receiving regular follow-up services or have a lot going on. They don’t get the form. It’s to protect the parents a bit, not rub it in that their child is abnormal. But things can easily slip through, since it’s the administrative staff who send it out. So, I must be attentive (…) They might slip, and suddenly it’s sent out, and then you have distressed parents calling. That’s not good.

This statement illustrates the PHNs’ underlying sense of responsibility to shield parents from the potentially reductive or normative lens of the screening, reflecting their concern for protecting the trust and relational safety that underpin their work. Simultaneously, participants expressed a need to maintain control over who should receive the screening, as a way of safeguarding the relationship with the family. A similar form of control appeared in relation to children, where some PHNs sought to protect them from conversations that might feel burdensome or portray them negatively.

Where I see high responses outside the normal range in hyperactivity or behavioral challenges, we can discuss it. It depends on the child, right. (…) We should phrase things in a way that doesn’t cast the child in a negative light. (…) Then there’s often a follow-up dialogue one-on-one with parents, right” Participant 003.

PHNs expressed their own professional judgements by assessing when structure fits and when they need to be adapted to the family’s situation to protect both children and parents. Embedded in this was a perception that standardized practices do not fully capture the complexity of family encounters, and that professional care therefore involved standing between the demands of structure and the need of the relationship. Being good, PHNs here appeared as finding a balance between following guidelines and exercising judgment to safeguard the child and the parents. Simultaneously, this position was time-consuming and difficult to maintain, especially as the tools within the structures increasingly determine who receives what, and as automated solutions risk constraining the scope for professional discretion.

### When we get trapped within the structures

This theme concerns how PHNs expressed being trapped within the structures. They perceived the screening as something imposed by management and the project, as something they had to use to do their job. Simultaneously, the screening generated information they did not know how to interpret and handle, leaving them uncertain about appropriate follow-up, which was reinforced by lack of support.

The PHNs expected the screening to provide them with objective information about the child’s situation. However, they experienced that the results often did not align with their own observations or conversations with parents. Participant 011 explained that this experience undermined their trust in the information provided by the screening.

I don’t feel I can trust the results. I don’t experience it as an objective measurement tool that provides trustworthy information. If the result is good, I can’t be sure it is truly good. If it is poor, I can’t be sure it is truly poor.

This created frustration as PHNs navigated the tension between their own observations and screening results. Rather than using both as complementary perspectives, the information was positioned against each other in structures that demanded clear right-or-wrong answers. Physical challenges were typically addressed with practical advice or referral, whereas psychological challenges were often normalized as age- and context- appropriate. PHNs expressed that physical issues were easier to categorize, whereas psychological concerns required more interpretation. When scores on mental health (Strength and Difficulties Questionnaire), quality of life (KIDSCREEN), and socio-emotional development (Ages and Stages Questionnaire- Social and Emotional) suggested difficulties, PHNs’ judgments of what was normal were challenged by a screening result that appeared objective. This reduced confidence in their professional assessment and increased uncertainty, partly because they lacked sufficient knowledge about the screening. Participant 005 explained: *“Yes*,* or more training*,* so we understand what we are answering. And… yes… something like an action plan if they score high here and there*,* what do we do then?”.*

Faced with this uncertainty, the PHNs asked for more guidelines, which contrasted with their earlier wish to focus on the relationship with families. They wanted guidelines to provide reassurance in complex situations, as they feared being held accountable now that screening scores were documented in the EHR. As participant 005 reflected during observation: *“They could suddenly come back and say*,* ‘why didn’t you do anything here—look at that score.’”*. This concern was particularly linked to psychological challenges. Consequently, what was considered doing a good job shifted from being present in relationship with families to adhering to the screening and doing what was deemed right within the screening tool’s logic.

The PHNs described uncertainty and fear when following up with children who scored in the borderline range on screenings addressing phycological challenges. They felt left alone with these assessments, lacking collegial support and without established routines for discussing results with other actors around the child, such as schools or kindergartens. They also experienced unclear referral pathways for these children. As Participant 003 explained:

Some children who score in the borderline range might not be identified without the screening, but it’s difficult. There’s nowhere to refer these children […] It’s hard to get information that this or that child isn’t doing well, and then I can’t do anything about it.

This created a sense of powerlessness and uncertainty about what fell within their mandate. They considered that their mandate was to guide parents, but not to deal with psychological challenges, and the boundaries between these tasks were perceived unclear. When children scored outside the normal range, follow-up was often understood as someone else’s responsibility. Parents’ reluctance to accept help for psychological challenges reinforced this experience. Whereas screening on physical difficulties could give PHNs confidence and authority to legitimize concern and propose measures, the situation was different when not being confident about the meaning and implications of screening results. Because they did not trust the results from the screening, they placed greater emphasis on parents’ perspectives in conversations, and responsibility for follow-up was shifted to parents or the school or kindergarten staff. This hesitation to override parents’ perception of whether there was a problem was captured by participant 011, who said:

If they don’t see it as a problem, you can’t just pile a lot of things on them. If they feel it’s manageable, then that’s how it is. And if the kindergarten sees it as an issue, then they should act and call a meeting from their side.

For PHNs, this felt like being caught between conflicting demands: a strong sense of responsibility for the child and family, but limited capacity to act. Simultaneously, their experiences were illustrated by lack of confidence in their own competence and in their ability to acknowledge challenges and explore them together with the family. Not because they were unwilling to help or support, but because they were navigating a demanding balance between their experience of the expectations from the structures, their experience of absence of referral options, and their eagerness to respect the family’s autonomy.

### When the structures support our practice

This theme serves as a contrast to the other themes in PHNs’ tensioned experiences, and concerns with how online screening tools were experienced to enhance relational practice among PHNs, fostering better interactions with families and interdisciplinary collaboration.

PHNs acknowledged that using online screening tools could be time-consuming yet emphasized how the structure provided by the tools helped them prepare in advance and offered a clear starting point for conversations with families. This structure enabled PHNs to address important topics more directly and efficiently. Participant 003 explained how the tool opened dialogues that might otherwise be avoided: *“The screening leads you into a dialogue you wouldn’t have had otherwise. It opens a conversation about something that might be private*,* shameful*,* or embarrassing.”*

While some PHNs felt compelled to follow the guidelines strictly and collect missing information to cover every point in both the national guidelines and the screening, others adopted a more flexible approach, prioritizing what mattered most to families, even if that meant not checking every box. Rather than feeling constrained by the tools, these PHNs described how they enhanced practice by shifting the dynamic from directive advice to participatory dialogue, fostering collaboration as families reflected on their child’s development. This shift encouraged greater user involvement in assessments, as participant 008 explained: *“I think it influences the guidance in a good way*,* so that I might be a bit better at not telling them what they should do but instead allowing for more user involvement.”*

These reflections by PHNs illustrate an experience of transformation towards a more collaborative approach with families driven by both the PHNs’ preparation and the engagement of parents through the screening process. While some PHNs questioned the objective of the screening tools, others highlighted how the tools did not give them an unequivocal answer, but a perspective from the parents. A perspective they could use to open a dialogue and contribute to a more balanced, reflective, and user centered approach. PHNs felt enabled to tailor their guidance to the family’s context while reinforcing the relational aspect of their practice, which they experienced as strengthening their clinical judgment and improving the quality of well-child visits. Participant 002 explained how the tool helped validate and sharpen their assessments: *“You see what the parents have answered*,* and then you make your own assessments while sitting there. I think using it helps ensure better quality in the well-child visit.”*

In addition to supporting individual assessments, the tools were experienced to offer insight into the child’s functioning across different contexts and were here used in collaboration with other services. Observation data revealed examples where PHNs obtained consent from parents to contact the kindergarten and asked them to complete the screening. Also here, discrepances were not seen as problematic, but rather as openings for professional curiosity and tailored intervention. Participant 004 described how diverging scores became a starting point from both dialogue and actions:

He scored higher on emotional difficulties in kindergarten than at home. They had very different perceptions of the child. So, we had to explore why it was harder for him there. (…) Then we could implement specific measures and later, evaluate.

This quote illustrates an experience of established routines for collaboration with kindergartens and a structured approach to follow-up. PHNs also described established routines for discussing screening results with colleagues and low-threshold practices for discussing scores with specialist health services when uncertain. Together, these routines represented a clear shift from PHNs’ earlier experiences of limited support.

Furthermore, the screening tools were seen as more than technical instruments; they functioned as communicative resources that offered a shared language both with parents and with other actors involved in the child’s care, while also providing concrete documentation for cross sector collaboration. This was essential in referral processes, where standardized formats transformed subjective impressions into tangible evidence, thereby strengthening the legitimacy of professional assessments and facilitating trust between services. As participant 004 explained: *“Instead of writing a note saying I think the child is like this or that*,* I can show that this has been done and observed. It’s a tool many are familiar with*,* so it becomes something concrete.”*

## Discussion

Findings from this study illustrate how PHNs faced tension between structural demands and relational priorities when online screening tools were introduced into their practices. Relationships with families were seen as the cornerstone of their work, yet the screening tools could both support and challenge this foundation. To further understand this tension, we will discuss the findings in light of the theory of acceleration and resonance presented by Rosa (2024). Rosa [27] describes modernity as driven by acceleration, a continuous increase in the pace and rate of change in technology, society, and everyday life. This dynamic creates pressure that can lead to alienation, where individuals lose a sense of connection and meaning in their engagement with the world. As a counterpoint, Rosa [27] conceptualizes resonance as a responsive relationship between individuals and their world, where openness to being affected by the encounter may lead to mutual, unpredictable transformation. Resonance emerges when we experience being in dialogue with the world and with others, whereas alienation occurs when interaction becomes instrumental and governed by external demands [27]. In the following, the discussion will focus on how the logic of acceleration, with its emphasis on efficiency and structure, influences the possibility for resonance in professional encounters, and how PHNs navigate this tension between structure and relational care.

Online screening tools can create conditions that foster resonance in PHNs practice. By offering structure and opportunities for preparation, they enabled both parties to enter the well-child visit with greater presence. Because many questions had already been considered in advance, the visit could begin with dialogue rather than routine tasks, reducing the initial uncertainty that often marks the start of an encounter. This predictability supported emotional safety, making it easier for parents to be open and engaged, which is consistent with research showing that screening only becomes meaningful when followed by a supportive, relationally attuned dialog [15, 28]. In this way, the tools did not replace relational work but created conditions that allowed it to unfold more fully, and those also supported moments of resonance, as both PHNs and parents met with greater openness and time.

Previous studies have shown that pre-visit screening helps parents articulate observations and concerns they might otherwise hesitate to raise [5, 29]. This pattern was also evident in the present study, where PHNs observed that parents became more aware of their child’s development and the everyday factors influencing it. This heightened awareness provided a stronger foundation for jointly exploring concerns rather than relying on directive guidance, aligning with parent-reported experiences in earlier research [16, 30]. The online screening tools also offered a shared framework and language for discussing these reflections, strengthening collaboration not only with families but also across agencies. As the conversation shifted from one-way instruction to a more collaborative process, the interaction became increasingly mutual. Several studies highlight that screening tools can support exploratory dialogue when health professionals have the time, responsiveness, and confidence to use them flexibly [2, 31]. An important part of this exploratory work involved attending not only to concern but also to the strengths that emerged through the screening tools. Discussing these strengths can reinforce trust, support a more balanced relational dynamic, and help parents feel recognized as competent caregivers, further deepening the collaborative quality of the encounter [16, 31]. In these ways, the use of online screening tools strengthened the relational quality of the encounter and supported conditions for resonance, as the dialogue became more open, mutually engaged, and responsive [27].

Simultaneously, the structuring effect of screening tools can, under certain conditions, shift practice toward acceleration and instrumentalization, limiting the openness that resonance requires. Time pressure following the implementation of online screening tools emerges, both in this study and in previous research [6], as a key challenge. When attention is redirected from the child and parents to managing digital inputs, the interaction risks becoming procedural rather than dialogical. Moreover, PHNs experienced that the tools did not fully cover the requirements set by national guidelines, which resulted in additional workload. Drawing on the theory of acceleration presented by Rosa [27], this illustrates the paradox of acceleration: attempts to streamline and ensure quality generate an increased volume of information requiring follow-up, thereby intensifying workload rather than reducing it. PHNs’ call for more comprehensive screening tools reflects a demand for dynamic stabilization, seeking structural solutions to maintain control, which, paradoxically, may reinforce the acceleration instead of fostering resonance. This aligns with previous research emphasizing that successful implementation must build on and interact with existing practices [32].

Furthermore, PHNs reported that the screening tools created a sense of distance from parents already during their distribution. They expressed particular concern that vulnerable families might feel stigmatized or judged, and that the relationship could get damaged. Rosa [27] refers to this as alienation, when interaction becomes subordinated to systemic demands for efficiency and control, resonance between actors is weakened, and the relationship loses its intrinsic value. To counter this and regain a sense of control, some PHNs chose not to send forms to all families, to preserve closeness and relational quality. When examined in relation to previous studies, this raises an important dilemma. Previous studies indicate that implementing screening tools does not guarantee universal coverage, and disparities across demographic groups may persist [33]. Likewise, literature on pediatric clinicians reveals that they are less likely to initiate discussions on sensitive topics, such as mental health or family stress, with the most vulnerable families [34]. As previous research suggests, selective practices aimed at preserving trust can paradoxically reinforce disparities, limiting access for vulnerable families. In other words, the intention to protect relational quality may unintentionally reduce engagement and prevent those most in need benefiting from these tools.

Moreover, both in the current, and in previous studies [35], PHNs described difficulties trusting parents’ responses and noted discrepancies between screening results and their own observations. Many operated with an underlying assumption that screening provided objective facts about the child’s situation. In contrast, other PHNs viewed the screening results not as objective truth, but as one perspective. When the screening was used as an entry to dialogue, this could be a strength; however, it caused insecurity when they expected definitive answers. In Rosa’s [27] perspective, this tension challenges resonance: the relation demands interpretation and context, while the acceleration logic values measurability and standardization. In this study, PHNs found physical challenges easier to understand and handle than psychological challenges. Given the acceleration logic, physical challenges were easier to measure because the child either met the expected milestone or not, making them appropriate for standardized assessment in an online tool. Psychological challenges, however, are dynamic and context-dependent, and require understanding over time and through dialogue [3]. As a result, psychological assessments became more demanding in accelerated practice, where efficiency and standardization were prioritized.

This perceived insecurity was also connected to insufficient knowledge about interpretation and managing these tools. Similar patterns have been documented elsewhere, showing that limited competence among healthcare professionals can influence decision-making and reduce confidence in clinical practice [6]. Additionally, previous studies found that PHNs lack the theoretical and evidence-based knowledge needed to identify and evaluate psychological difficulties [3]. In structures that prioritize efficiency and standardization, these competence gaps exacerbate uncertainty and make it particularly challenging to capture the nuanced, context-dependent nature of psychological assessments. Targeted training on the purpose of the screening tools and how they can be meaningfully integrated into everyday practice may help reduce this uncertainty [11]. When training and support fail to keep pace with rapid changes, tools intended to support practice were instead experienced as obstacles, contributing to feelings of alienation [27]. This sense of vulnerability was further intensified by fears of being accused of not having done enough. Digitalization increases the visibility and traceability of documentation, and when professional practice must be legitimized primarily through screening data, a rupture in resonance occurs. Relationships risk losing their intrinsic value and becoming overshadowed by the demand to document completed tasks.

Additionally, several PHNs experienced a lack of support and limited options for referrals, especially for children who scored in the borderline area. These circumstances can be seen as experiences that weaken PHNs’ resonance with the structures, resulting in a feeling of being trapped or isolated. Their felt structural isolation reduced both the ability to act and the sense of relational safety, while the structure’s focus on efficiency and control overshadowed the value of collaboration. In contrast, PHNs who experienced having easy access to collaboration services and felt supported described a relationship with the structures that were more responsive and supportive. This gave them greater freedom to act and a stronger sense of safety in their professional role. For PHNs who face insecurity and lack of support, some PHNs attempted to regain control by either avoiding the use of screening results during well-child visits or shifting responsibility to parents, kindergartens, or schools, depending on whether they perceived the child’s challenges as problematic. As a result, the focus shifts from the child’s needs to a question about responsibility. When PHNs avoid discussing screening results during well-child visits or transmitting the responsibility, this practice risks undermining their mandate to promote early detection and provide guidance based on the child’s best interests [19]. This avoidance and shifting responsibility can reinforce feelings of vulnerability and uncertainty among parents, which in turn limits the potential of online screening tools to support families [15, 17]. These reactions can be understood as a form of deceleration [36]. Resistance represents an attempt to protect professional autonomy and relational values. The shifting of responsibility can be seen as a strategy to avoid further acceleration. Simultaneously, PHNs’ skepticism can be seen as an expression of the limits of flexibilization: the profession is expected to adapt to new demands for speed and documentation, yet this collides with the stable core tasks of practice. In Rosa’s [36] terms, this signals that acceleration is beginning to challenge its own institutional and cultural foundations.

PHNs who experience insecurity, limited support, and lack of referral options often express a desire for clearer guidelines. Clearer guidelines have also been suggested in previous studies as a strategy to reduce uncertainty and ensure follow-up for children with positive screening results [11]. However, we argue that additional guidelines alone do not resolve the underlying challenge. Under pressure from acceleration, professionals seek frameworks that provide stability, define responsibilities, and protect against further acceleration of their work. Guidelines and structure can be understood as stabilizing mechanisms in a context where digitalization and new requirements threaten professional flexibility [37]. While they offer security and predictability, they may also restrict dialogue and professional judgment, thereby limiting resonance. This creates a paradox in PHNs’ experiences: they seek structure to cope with growing pressure, while valuing presence and relationship, elements increasingly challenged by standardization. The tension between acceleration and resonance becomes clear: the need for stability can undermine what gives meaning to professional practice.

The Norwegian Directorate of Health [19] already provides explicit recommendations and established referral routines. PHNs are responsible for promoting health, assessing children’s development and well-being, supporting and providing guidance to parents, and initiating referrals when needed in collaboration with other services. Consequently, additional guidelines alone were unlikely to resolve the problem. Additionally, implementations studies also often recommend co-design to ensure that new solutions resonate with practitioners’ values and everyday work [38]. Co-design is therefore frequently used as a reference point when examining why certain tools fit well in practice while others create friction. Although the program was developed through a co-design process involving PHNs [20], our findings indicate that several of the tensions they experience in practice extend beyond the online tools themselves. These tensions reflect deeper structural and relational dynamics already present in the service, suggesting that co-design, while valuable, cannot fully compensate for systemic pressures, organizational constraints, or the emotional and relational demands of the work. Moreover, new tools or procedures cannot always be expected to fit seamlessly into existing workflows, as effective implementation requires changes in professional practice and organizational support systems [18].

Based on our discussion, we suggest that measures combining stability with resonance may be more effective. Such measures should include strengthening PHNs’ confidence and competence in online screening tools and addressing sensitive topics, while ensuring access to collaboration and professional support so they can manage demanding situations without compromising relational trust. Giving space to, and invite PHNs, to reflect on what resonates with them, how they can feel affected, and how they affect others (families), would also be crucial to maintain in relationship with their professional values and be grounded in humanity as carers. Guidelines should be principle-based and allow professional judgment, and online tools should be integrated in ways that preserve relational time rather than reduce it. As we argue here, structure and relationships are not opposite; they need to be balanced within an accelerated healthcare context. Instead of abandoning online screening tools, they should be embedded in ways that foster dialogue and resonance, ensuring systemic demands do not overshadow relational value. This balance may help manage pressures of acceleration without eroding the profession’s core values.

Participation in this study was limited to PHNs who had used the tool, so perspectives among non-users were not explored. Moreover, findings reflect a specific context, shaped by factors such as workplace culture, leadership, resource availability, technological infrastructure, and policy priorities, dimensions beyond the scope of this study. Further research is needed to explore how more resonant practices can be implemented in practice. This includes examining organizational support and professional development initiatives that promote safe and sustainable integration of online screening tools. Another limitation is that most of the data collection was conducted before the platform update, at a time when PHNs had more limited experience with use of online screening tools. As the updated platform introduced changes to usability and workflow integration, PHNs’ experiences may have evolved over time. It is therefore possible that some of the concerns or benefits identified in this study would appear differently if data were collected after the update, when the tool was more stable and PHNs had gained greater familiarity with its use. Future studies should also include the family perspective to examine how screening affects parents’ experiences, trust in health services, and family dynamics, and assess long-term outcomes for children who are identified and followed through these tools.

## Conclusion

This study highlights tension between structural demands for standardized assessment and the relational foundation of PHNs’ work when online screening tools were integrated into their practice. On one hand, they introduced uncertainty and vulnerability related to interpretation, follow-up, and professional mandate; on the other, when PHNs felt confident and supported, the tools were perceived as supportive for practice and as a means to strengthen what was seen as the cornerstone of the PHNs work, the relational dimension of care. The findings suggest that online screening tools shape professional routines and relational dynamics in ways that require careful consideration. Understanding these experiences is essential for informing future strategies that respect both structural requirements and the relational values central to child and family health nursing.

## Electronic Supplementary Material

Below is the link to the electronic supplementary material.

Supplementary Material 1

## Data Availability

The datasets used and/or analyzed during the current study are available from the corresponding author on reasonable request.

## Declarations

### Use of AI tools

Language editing and editorial improvements were partially supported by a large language model tool (Microsoft Copilot, Microsoft). The model was used to suggest linguistic refinements, but all changes were reviewed and approved by the authors.

## Abbreviations

ASQ: Ages and Stages Questionnaire
ASQ-SE: Ages and Stages Questionnaire- Social Emotional
EHR: Electronic health record
PHN: Public health nurse
SDQ: Strength and Difficulty Questionnaire
