# Supplementary material for: Navigating structural demands and relational care: a qualitative study of public health nurses’ experiences and handling of online screening tools
Source: BMC Nurs. 2026 May 7;25:580. doi: 10.1186/s12912-026-04727-4 (PMC13321622; doi:10.1186/s12912-026-04727-4)
Supplement: Supplementary file 1 — Supplementary Material 1 [file 12912_2026_4727_MOESM1_ESM.docx]

## Interview and Observational Guide

(Translated from original Norwegian version)

### Information Provided to Participants Before Observations

Brief oral information will be given about the study and the think‑aloud method.

Participants will be informed:

“Tell me everything you do and think. Don’t worry about planning what to say or clarifying your thoughts. I want to hear your thinking continuously, unfiltered and uninterrupted.”

“If you need time to think, that’s fine—just tell me what you were thinking about as soon as possible.”

“If you are silent for too long, I will say ‘please keep talking’ to remind you to think aloud.”

“This is an exploratory study. My intention is not to evaluate your thinking or explanations, but to learn about your thought processes as you carry out your tasks.”

Participants will also receive a demonstration/explanation of the audio recording process. Information about consent, the right to withdraw, confidentiality within the project, and mutual confidentiality between participants will be repeated.

### General Prompt for Observations

“Can you continue thinking aloud?”

“What do you think about this situation?”

“Can you elaborate on your reasoning?”

### Observational guide

#### Preparation Phase

Where do they retrieve information from? How easy is it to find?

What is the perceived usefulness of the information presented?

How do they use the information during preparation?

Which information is emphasized in their assessments?

How do they interpret the information/results before the well-child visit?

What is different now compared to before using parent‑completed online screening tools?

#### During the Well-Child Visit

How is the available information used—or not used—during the well-child visit?

Do the findings in the well-child visit align with the screening responses?

Which information is emphasized during the well-child visit?

How do the online screening tool influence communication between parents and the public health nurse?

When accessible: Are screening data used during the well-child visit?

If so, how are online screening results communicated to families?

Do families bring up the screening themselves?

#### Post-visit tasks

Which information is emphasized in decisions made after the well-child visit?

Is information from the online screening tool used?

Are additional actions or interventions initiated based on screening results?

Collaboration with colleagues or other partners: Which information is emphasized?

What is documented after the well-child visit, and where is it documented?

Who has access to the information, or who is it forwarded to?

### Interview Guide

#### Introduction

Brief oral information about the study. Demonstration/explanation of audio recording. Information about consent, the right to withdraw, confidentiality within the project, and mutual confidentiality. Expected duration: approximately 45 minutes.

#### General Probing Questions

“When you say…, do you mean…?”

“Is it the case that…?”

“In other words, can we say that…?”

“How do you think about that?”

“In what way do you mean…?”

“What do you think contributes to…?”

“What do you think would happen if…?”

#### Introductory Questions

Age

Education

Employment status

Experience as a public health nurse

Experience with questionnaires

Which questionnaires have you used?

#### Main Questions

Can you describe a typical process for a well‑child visit?

- Preparation
- The well-child visit
- Post-visit tasks
- Information handling
- Interaction between nurse and parents/guardians and their child
- Assessments and decisions—based on what information?

How has this process changed after the implementation of online screening tools through Starting Right?

- Preparation
- The well-child visit
- Post-visit tasks
- Information handling
- Interaction with parents/guardians and their child
- Assessments and decisions—based on what information?
- Changes related to finding and using available information
- Changes related to documentation routines and information sharing

3. Can you describe your experiences using the *Starting Right online* screening tool?

- Useability
- How is the information presented?
- Whether it supports and improves decision‑making when assessing children
- Examples of how you use the screening results to make decisions
- Relevance of the content in relation to: retrieving clinical reports and alerts about child health, early identification of children needing additional follow‑up.
- How the use of screening influence interaction with parents and their children
- Use of collected health data in follow‑up of children
- Use of collected health data in collaboration with physicians/physiotherapists or other actors

#### Closing

Is there anything else you would like to add?

Thank you for participating

Permission to contact you again?

Reminder about confidentiality
